# Fracture strength and failure patterns of different restoration approaches in endodontically treated maxillary premolars: an in vitro study with fractographic analysis

**DOI:** 10.1186/s12903-026-08064-5

**Published:** 2026-03-27

**Authors:** Amira Yehia Amin, Amina Hamdy, Marwa Wahsh, Amr El Etreby, Marwa Emam

**Affiliations:** 1 Department of Fixed Prosthodontics, Faculty of Dentistry, Ain Shams University, African unity street, El-Khalyfa El-Mamoun Street Abbasya, Cairo, Egypt; 2 Department of Fixed Prosthodontics, Faculty of dentistry, Galala University, Galala Plateau, Attaka, Suez Egypt

**Keywords:** Fiber Post, core, and crown, Endocrown, Overlay, Endodontically treated premolar, CAD-CAM, lithium disilicate, bulk fill composite, Fracture strength

## Abstract

**Background:**

The restoration of endodontically treated premolars has been a controversial topic. Whether a post or a more conservative option should be used remains inconclusive. The present in vitro study aimed to evaluate the fracture strength and failure pattern of different partial coverage restoration approaches for endodontically treated upper premolars in comparison to the post, core and crown approach.

**Methods:**

Thirty-five maxillary premolars were endodontically treated and assigned to five groups (*n* = 7): endocrown (E), endocrown with buccal veneer (EB), overlay (O), overlay with buccal veneer (OB), and post, core, and crown (P). All restorations were fabricated from lithium disilicate ceramics and subjected to vertical static loading until failure. Fracture strength was recorded, and failure patterns were examined using a stereomicroscope and scanning electron microscopy. Data were analyzed with Welch one-way ANOVA and Games–Howell post hoc test (α = 0.05).

**Results:**

Significant differences were found among groups (p < 0.001) with a large effect size (partial eta squared = 0.83 (95% CI; 0.59 to 0.91). The highest mean fracture strength was observed in group P (1676.29 ± 191.25 N), followed by OB (1475.00 ± 198.72 N), E (1374.71 ± 371.91 N), EB (1047.41 ± 163.90 N), and O (987.99 ± 125.01 N). Groups P and OB demonstrated significantly higher strength than EB and O. Group P exhibited only repairable restoration fractures, whereas the other groups predominantly showed catastrophic failures.

**Conclusions:**

Within the limitations of this in vitro study, post, core, and crown restorations provided superior fracture strength and the most favorable failure patterns under static axial loading. While overlays with buccal veneers demonstrated promising mechanical resistance thresholds, their clinical application should be considered with caution. These designs may serve as a potential conservative alternative, although further research involving dynamic fatigue and oblique loading is required to confirm their long-term clinical durability in the complex oral environment.

**Clinical relevance:**

The findings of this in-vitro study provide insight into the fracture strength and failure tendencies of different restorative approaches for endodontically treated maxillary premolars. While post, core, and crown restorations demonstrated high load-bearing capacity and predominantly favourable failure patterns, overlay designs with a buccal veneer showed comparable strength but a greater tendency toward catastrophic fractures. These observations highlight a potential balance between structural preservation and failure reparability. Given that static axial loading was used, direct clinical interpretation should be approached cautiously. Treatment planning should integrate these biomechanical considerations with patient-specific factors, functional demands, and clinical judgment when selecting restorative approaches.

## Background

 Restoration of severely damaged coronal hard tissue associated with endodontic treatment has been a challenge in reconstructive dentistry [1, 2]. Endodontically treated teeth (ETT) suffer a reduction in stiffness and fracture strength due to loss of structural integrity associated with decay, preparation, and the whole sequence of root canal treatment, rather than dehydration or physical changes in the dentin [1]. Endodontically treated upper premolars, in particular, have a greater risk for biomechanical failure owing to their occlusal anatomy which is characterized by accentuated cusp inclinations. In addition to the narrow cervical thickness they have, which play a crucial role in the distribution of masticatory forces, with vertical root fractures often initiating and spreading from this area [2].

For many years, post and core systems have been used as foundational materials for the final restoration of endodontically treated premolars that have lost most of their coronal tooth structure [3]. The development of adhesive techniques, ceramic materials, and digital dentistry was a genuine breakthrough in the restoration of ETT. With adhesive restorations, a macro-retentive design is no longer a prerequisite if there are sufficient tooth surfaces for bonding [4, 5]. Restoration techniques with post-free and core build-ups are growing in popularity due to their minimal invasiveness and simplification of clinical procedures [6]. Partial coverage restorations can provide the required protection and ensure clinical success of the restoration while providing the maximum preservation of tooth structure [7]. These restorations may be divided into three categories based on the amount of cusp coverage: inlays, where there is no cusp coverage, onlays, where at least one cusp is covered, and overlays, where all cusps are covered [8].

Endocrowns on the other hand, bring together the intraradicular post, core and crown in one unit, where the pulp chamber is used to improve retention through a wider surface area for adhesion, thus increasing stability [9]. The incorporation of a buccal cervical margin in partial-coverage restorations for endodontically treated maxillary premolars has been advocated in the literature to address esthetic limitations associated with preparations confined to the occlusal third. Positioning the margin within the cervical third, close to the gingival line and within the esthetic hidden zone, allows for improved masking of restorative interfaces and enhanced optical integration. Although this design necessitates additional tooth reduction, it provides superior esthetic outcomes and more favorable margin concealment, which may justify the extended preparation in cases with high esthetic demands when executed with careful preservation of sound tooth structure [10].

Several laboratory studies and clinical trials have compared the performance of partial coverage restorations to full crowns in stress-bearing posterior teeth [11, 12]. Some studies have demonstrated that placing posts in endodontically treated maxillary premolars significantly enhances their fracture strength [13, 14]. Conversely, it has been reported that the resistance of endodontically treated maxillary premolars to fracture with and without posts was similar when other restoration approaches provided cusp coverage [15].

Given the inconsistency in the literature regarding the optimal restorative protocol for endodontically treated maxillary premolars, the present study aimed to investigate the fracture strength and failure pattern of different partial coverage restoration approaches: Endocrown, Endocrown with buccal veneer, Overlay, Overlay with buccal veneer in comparison to the conventional post, core and crown approach. The null hypothesis was that different restoration approaches would exhibit no significant differences in fracture strength or failure patterns.

## Methods

### Ethical clearance and study design

**Fig. 1:**
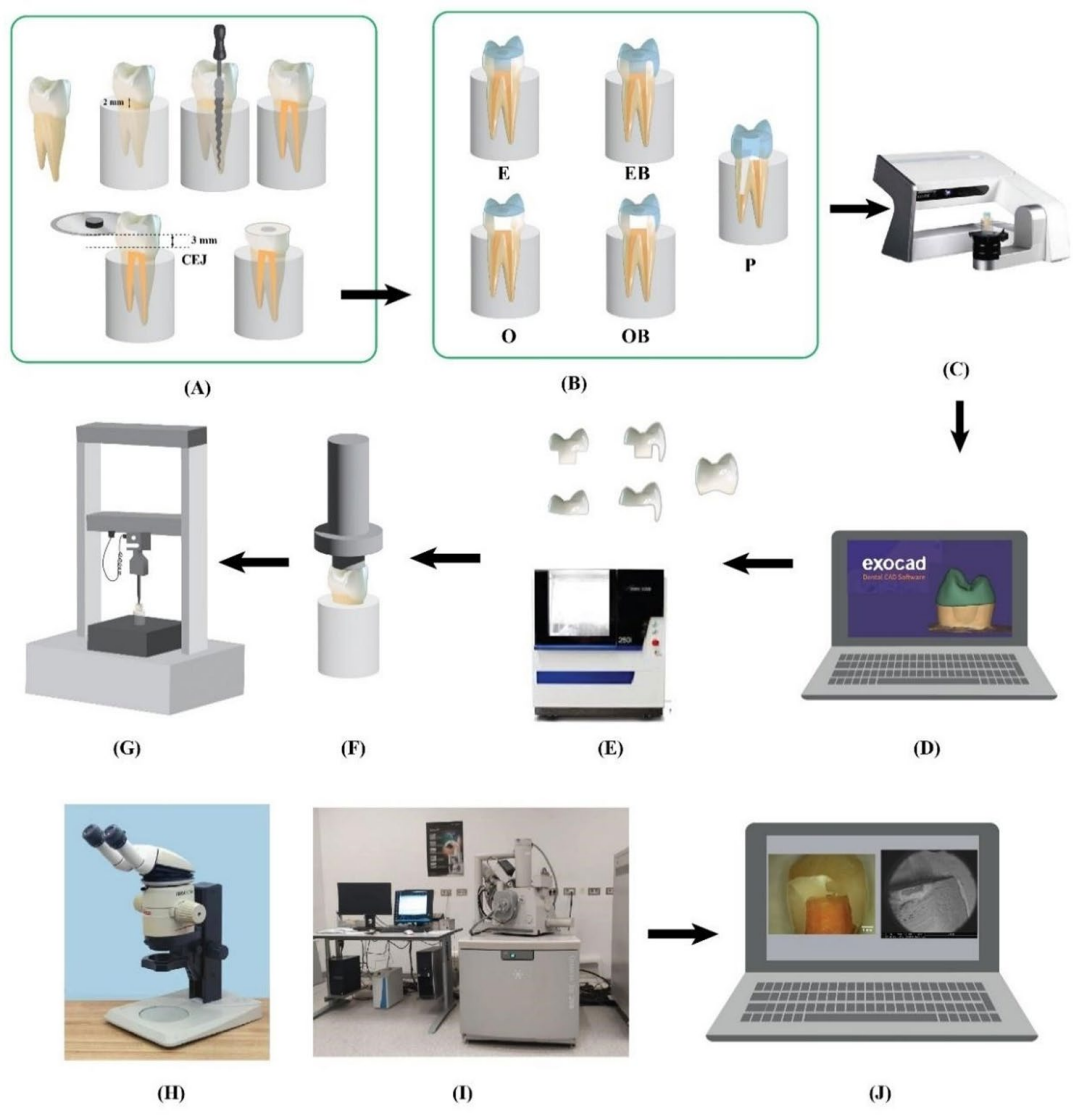
Study overview. **A**: Extracted upper premolars were placed in acrylic blocks, endodontically treated, and decoronated 3 mm above the cementoenamel junction. **B**: Samples were prepared with different preparation designs. **C**: Samples scanning. **D**: Designing restorations using dental CAD software and exporting STL files of restoration designs for milling. **E**: Restoration fabrication using CAM. **F**, **G**: Samples were vertically loaded after cementation in a universal testing machine. Imaging of failed samples using a stereomicroscope (**H**) and scanning electron microscope (**I**). **J**: Fractographic analysis performed for all samples

Research Ethics Committee of Faculty of Dentistry, Ain Shams University “FDASU-REC” waived the ethical approval for this study due to being an in-vitro study with no patients or animal experiments, and only anonymously extracted teeth were included. Endodontically treated upper premolars (*n* = 35) were categorized into 5 groups, which were restored as follows: endocrown (E), endocrown with buccal veneer (EB), overlay (O), overlay with buccal veneer (OB), and post, core and crown restorations (P). All restorations were fabricated of lithium disilicate ceramics, and all samples of all groups were vertically loaded till failure in a universal testing machine. The fracture load was recorded, and fractographic analysis was performed with a scanning electron microscope (SEM). The study overview is shown in Fig. 1.

## Sample size

Power analysis was designed to have adequate power to apply a statistical test of the null hypothesis that there was no difference between the different tested groups. By adopting an alpha (α) level of 0.05 (5%), a beta (β) level of 0.20 (20%), i.e., power = 80%, and using an effect size (f = 0.632) calculated based on the results of previous studies [16, 17]. Predicted sample size (n) was found to be a total of (35) samples, i.e. (7) for each group. Sample size calculation was performed using G*Power version 3.1.9.2 [18]. 

## Samples selection

A total of thirty-five freshly extracted, caries-free double rooted maxillary premolars, extracted for orthodontic purposes, were selected for the study. Teeth with anatomically average crown dimensions were included, based on bucco-lingual measurements of 9 ± 1 mm and mesio-distal measurements of 7 ± 0.5 mm. After assessment using a digital caliper, the selected specimens were subsequently cleaned and stored in distilled water at room temperature until further use.

## Periodontium simulation and teeth mounting

To simulate a 0.3-mm-thick periodontal ligament, each tooth root was coated in melted wax (Renfert, GmbH, Hilzingen, Germany) up to a demarcation line located 2 mm apical to the cementoenamel junction (CEJ). Teeth were then embedded in epoxy resin using a custom-made cylindrical sample holder (2 cm in length and 2 cm in internal diameter) and a parallelometer to ensure precise centralization. Embedding was performed up to the demarcation line on the proximal surfaces, simulating the bone level, and teeth were held in position until the resin had fully polymerized. Following polymerization, teeth were removed and placed in a warm water bath to soften the wax spacer. Subsequently, wax was eliminated, and a light-body polyvinyl siloxane material (Flexceed, GC South-East, Asia) was injected into the space between the mold and the root. Teeth were then reinserted into their respective molds.

## Endodontic treatment

All teeth were examined under ×20 magnification to exclude specimens with pre-existing cracks, fractures, or structural defects.

Standardized endodontic access cavities by an endodontic consultant were prepared using a #2 diamond round bur (Dentsply, Tulsa, OK, USA) followed by an ENDO-Z bur (Dentsply International, USA) in a high-speed handpiece under copious water cooling. K-files size 15 (Mani Inc, Japan) were introduced into root canals. By subtracting 0.5 mm from the length, the working length was recorded. ProTaper rotary instruments (Dentsply-Maillefer, Ballaigues, Switzerland) till master apical rotary size of F3 were used to prepare the canals. A lubricant (MD-Chelcream, METABIOMED, Korea) was used with irrigation using sodium hypochlorite 4.2% in between files, delivered with a side-vented needle after each instrument, with a total irrigation volume of approximately 10 mL per canal and a minimum contact time of 30 s per cycle. then after intermittent irrigation. all canals were finally rinsed with distilled water to eliminate any residual irrigants that could interfere with bonding procedures. The canals were then dried using sterile paper points prior to obturation.

Then, ProTaper F2 gutta-percha and AH Plus (Dentsply DeTrey, Konstanz, Germany) epoxy resinbased sealer was used for obturation. The root canals were filled with gutta-percha by lateral compaction technique using a #2 spreader (Mani, Japan) and #20 accessory cones. Excess gutta-percha was removed by a heated instrument at 0.5 mm below the cementoenamel junction (CEJ). The remaining gutta-percha was vertically condensed using a plugger [2].

### Samples preparation

All experimental groups were standardized in terms of tooth selection, root morphology, and endodontic preparation to ensure baseline comparability.

All specimens were sectioned 3 mm above the proximal cementoenamel junction to standardize occlusogingival height and preparation dimensions using a diamond disc under copious water cooling, then were stored in 0.9% sterile saline solution to prevent dehydration. Samples were then randomly divided into five groups (*n* = 7), according to the planned restorative approach.

In four of the five groups, conventional endocrown preparation was initially performed.

**Fig. 2:**
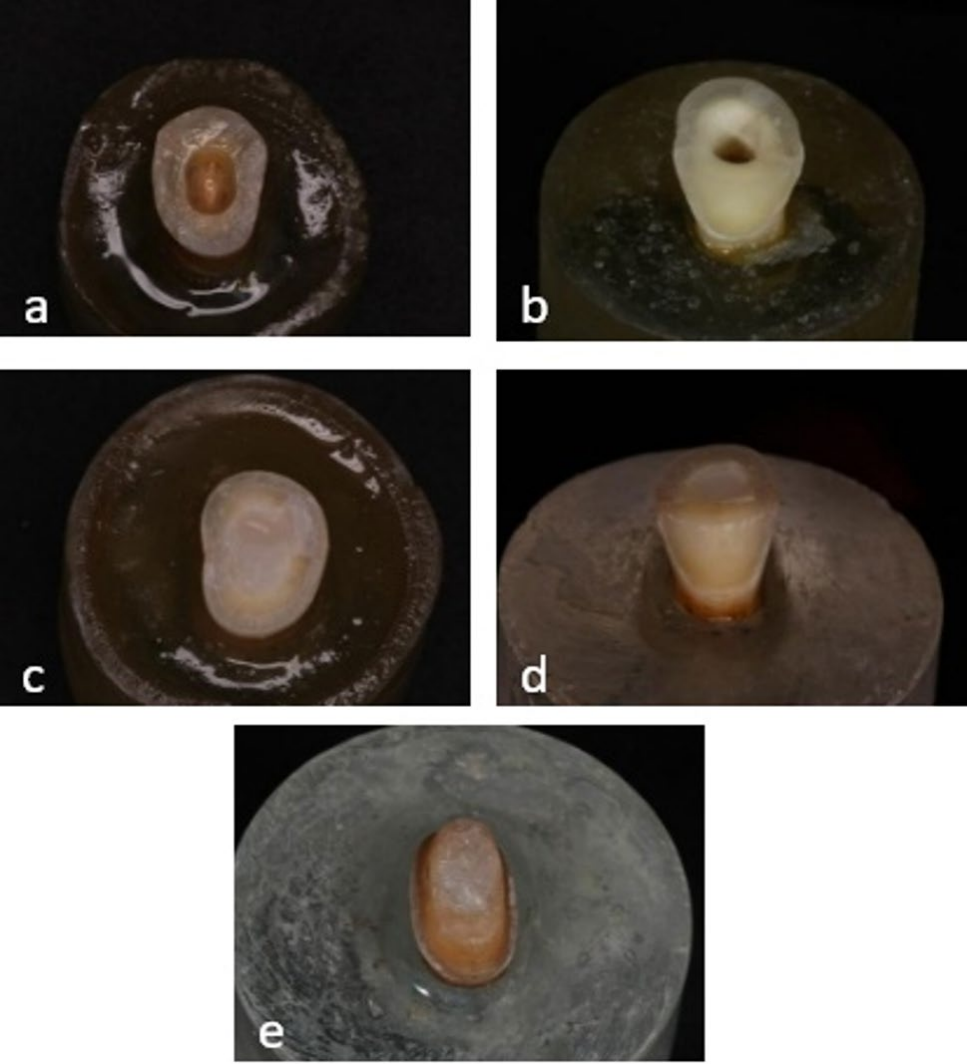
Different preparation designs; **A**: Endocrown, **B**: Endocrown with buccal veneer, **C**: Overlay, **D**: Overlay with buccal veneer, **E**: Post, core, and crown

Afterwards, three were further modified to produce the EB, O, and OB groups (Fig. 2).

#### Group E (Endocrown)

A traditional endocrown preparation was carried out by creating an internal cavity within the pulp chamber. Undercuts created during access opening and root canal instrumentation were removed and included in the endocrown preparation to standardize the internal geometry and provide a uniform path of insertion, and axial walls were aligned to achieve an internal taper of 8°–10° [19].

The cavity was prepared to provide a standardized circumferential butt margin with a wall thickness of 2 ± 0.2 mm. The depth of the intra-coronal cavity was standardized at 4 mm, measured from the internal cavity margin to the pulpal floor using a graded periodontal probe [20, 21].

#### In Group EB (Endocrown with buccal veneer restorations)

The same endocrown preparation as Group E was performed. Additionally, the buccal surface of the tooth was reduced to a thickness of 0.8 mm (confined to sound enamel) to allow for veneer application [10, 16].

#### In Group O (overlay restorations)

The pulp chamber was completely obliterated using a bulk-fill composite core material (SDR PLUS^®^, Dentsply International, USA), filled to the level of the tooth margin after adhesive application (Prime and bond universal, Dentsply International, USA) [9].

overlay design utilized in this study involved full occlusal coverage of both buccal and palatal cusps [22].

#### In Group OB (overlay with buccal veneer restorations)

Similar to Group O, the pulp chamber was obliterated with bulk-fill composite core material (SDR PLUS^®^, Dentsply International, USA), filled to the level of the tooth margin. Furthermore, the buccal surface was reduced to 0.8 mm to accommodate a veneer [10, 16, 23].

#### Group P (Fiber Post, Core, and Glass-Ceramic Crown)

Samples received glass fiber posts (Olident, Cologne, Germany) and bulk-fill composite core material (SDR PLUS^®^, Dentsply International, USA). Post spaces in palatal roots were drilled with # 4 Gates Glidden drills, leaving 4 mm of gutta-percha apically. Prepared post spaces were etched with 37% phosphoric acid (ETCH37 w/BAC, Bisco Inc., USA) for 15 s, rinsed thoroughly, and dried using compressed air and paper points [24].

A silane coupling agent (Porcelain Primer/Bis-Silane, Bisco Inc., USA) was applied to the post surface for 60 s and air-dried for 5 s before luting with a self-adhesive dual-cure resin cement (CALIBRA UNIVERSAL^®^, Dentsply International, USA) [25].

A dental surveyor (Af30, Nouvag USA Inc., USA) equipped with a straight handpiece mounted perpendicular to the surveyor platform was used for crown preparation and buccal veneer preparation following a standardized protocol. A 1 mm-wide circumferential deep chamfer finish line was established on sound tooth structure [25], preserving a 2 mm ferrule and providing an 8° coronal convergence for crown preparation, while for buccal veneer preparation, the tooth was reduced to a thickness of 0.8 mm to allow for veneer application with the same coronal convergence as for crown preparation.

## Restorations’ fabrication and cementation

**Fig. 3:**
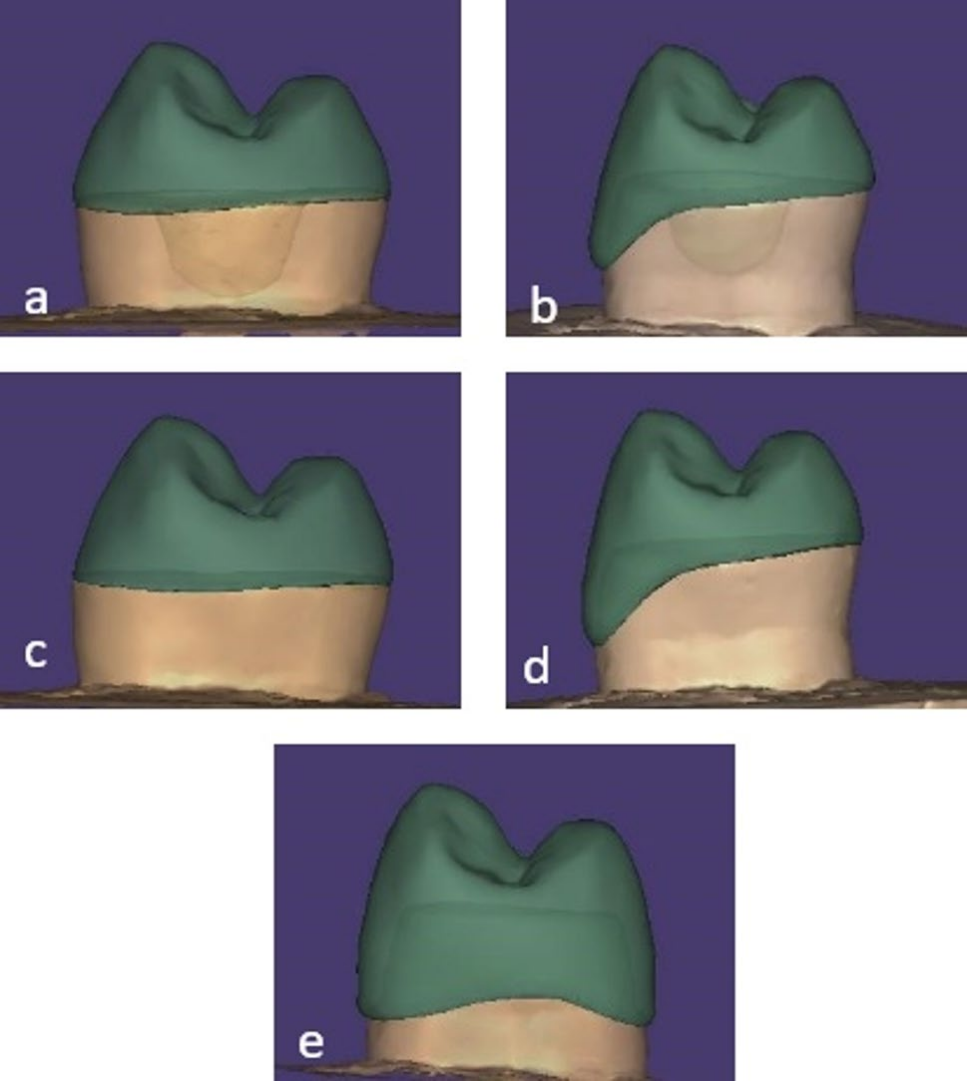
Different designs of the fabricated restorations; **A**: Endocrown, **B**: Endocrown with buccal veneer, **C**: Overlay, **D**: Overlay with buccal veneer, **E**: Post, core and crown

A desktop scanner (DOF Edge; DOF Inc., South Korea) was used to scan the samples and generate STL files. Restorations were then designed using Exocad software (ExoCAD Galway 3.0, Darmstadt, Germany) (Fig. 3). To standardize E.max CAD restorations, all lithium disilicate restorations were digitally standardized using the Biogeneric Copy mode in Exocad, ensuring uniform occlusal morphology across groups [3].

After the design process was completed, low translucency (LT) A1 lithium disilicate blocks (e.max CAD; Ivoclar Vivadent, Schaan, Liechtenstein) were milled using a CORiTEC 250i milling unit (imes-icore, Germany). A diamond bur was then used to separate the restorations and smooth their surfaces, followed by ultrasonic cleaning to eliminate any residual dust. Crystallization and glazing were performed using a compatible ceramic furnace (P300; Ivoclar Vivadent) at a temperature of 840–850 °C for 35 min following manufacturer instructions.

IPS e.max CAD restorations’ Intaglio surfaces were etched with 9.5% hydrofluoric acid gel (Porcelain Etchant Gel, Bisco Inc, USA) for 20 s, then rinsed for 60 s and dried for 30 s with moisture-free air. A ceramic primer containing silane coupling agent (Porcelain Primer/Bis-Silane™, Bisco Inc, USA) was applied and allowed to dry for 60 s.

Prepared tooth surfaces were etched using 37% phosphoric acid etching gel (Ultradent Product Inc., South Jordan, UT, USA) and abundantly washed and dried. Two separate coats of bond (All bond universal BISCO Inc, Schaumburg, IL, USA) were applied to the preparation, excess solvent was dried with oil-free air for 3 s, then light cured for 20 s at each surface. The dual-cure adhesive resin cement (DuoLink Universal, BISCO Inc., Schaumburg, IL, USA) was applied on the prepared surface of teeth, and restorations were bonded under a constant seating force of 3 Kg using a customized loading device, applied along the long axis of the tooth, to prevent restoration rebound until the cement fully polymerizes [24].

## Fracture load measurements

After water storage for 24 h, specimens were subjected to a vertical compressive loading in a universal testing machine (Model 3345; Instron Industrial Products, Norwood, USA) with a load cell of 5 KN, and data were recorded using computer software (Bluehill Lite Software, Instron^®^) [26–28].

Load was applied occlusally using a metallic rod with a round tip (3.6 mm diameter) attached to the upper movable compartment of the testing machine, traveling at a crosshead speed of 1 mm/min with a tin foil sheet in between to achieve a homogenous stress distribution and minimization of the transmission of local force peaks. The load at failure was manifested by an audible crack and confirmed by a sharp drop in the load-deflection curve. The load required to fracture was recorded in Newtons.

### Failure pattern

 While the study was sufficiently powered for the primary continuous outcome, caution should be exercised when interpreting categorical failure patterns.

After fracture strength test, specimens in the test groups were inspected visually in a blinded and calibrated manner under Leica MZ12.5 Stereo Microscope (Leica Microsystems, Wetzlar, Germany) to determine fracture pattern being favorable (repairable) or catastrophic (non-repairable), as illustrated in Fig. 5. Based on the extent and direction of fracture propagation relative to the cemento-enamel junction. Fractures were considered favorable if the fracture is repairable; either a tooth fracture above the cemento enamel junction or a fracture within the restoration while preserving the remaining tooth. catastrophic fractures were those with tooth fractures below the cemento-enamel junction (CEJ) or vertical fractures that are non-repairable [29].

### Fractography analysis

Fractographic evaluation of the fractured specimens was conducted using scanning electron microscopy (SEM) to identify failure origins and crack propagation features. Prior to SEM imaging, fracture surfaces were cleaned ultrasonically in ethanol to remove debris and were oriented in a standardized manner, with the tensile side positioned inferiorly and the compressive side superiorly, to facilitate consistent interpretation of fracture progression.

SEM (Model FEI Quanta 3D 200i) was used under operating conditions of accelerating voltage 30 K.V, resolution for Gun.1 nm, and different magnifications up to x2000 to examine fracture surfaces for characteristic fractographic markers including compression curls, fracture mirrors, and hackle patterns. These features were used to trace crack paths back to their origin following established qualitative principles of dental fractography. Finally, photomicrographs were recorded, and fracture morphology was analyzed according to the description and methods employed for brittle materials [30].

### Statistical analysis

Categorical data are presented as frequency and percentage values and were analyzed using Fisher’s exact test, followed by pairwise comparisons using multiple z-tests with p-value correction using the False Discovery Rate (FDR) method. Numerical data are presented as mean and standard deviation (SD)values. They were explored for normality and variance homogeneity by checking data distribution as well as using the Shapiro-Wilk and Levene’s tests, respectively. Data were normally distributed; however, the homogeneity assumption was violated, so they were analyzed using Welch one-way ANOVA followed by the Games-Howell post hoc test. The significance level was set at *p* < 0.05. Statistical analysis was performed with R statistical analysis software version 4.4.3 for Windows (R Core Team, 2025) [31].

## Results

There was a significant difference between the different groups (*p* < 0.001). The highest mean strength was found in P (1676.29 ± 191.25) (N), followed by OB (1475.00 ± 198.72) (N), then E (1374.71 ± 371.91) (N), and EB (1047.41 ± 163.90) (N), while the lowest strength was found in O (987.99 ± 125.01) (N). Post hoc pairwise comparisons showed that OB and P had significantly higher strength than EB and O (*p* < 0.001).

Intergroup comparison and summary statistics for fracture strength (N) are presented in Table 1.

**Table 1:** Intergroup comparison and summary statistics for fracture strength (N)

Fracture strength (*N*) (Mean ± SD)					F-value	*p*-value	PES (95% CI)
E	EB	O	OB	*P*			
1374.71 ± 371.91^AB^	1047.41 ± 163.90^B^	987.99 ± 125.01^B^	1475.00 ± 198.72^A^	1676.29 ± 191.25^A^	**18.57**	< 0.001*	**0.83 (0.59 to 0.91)**

*PES* Partial Eta Squared, *CI* Confidence interval, Values with different superscript**s** are significantly different; * significant (*p* < 0.05)

**Fig. 4:**
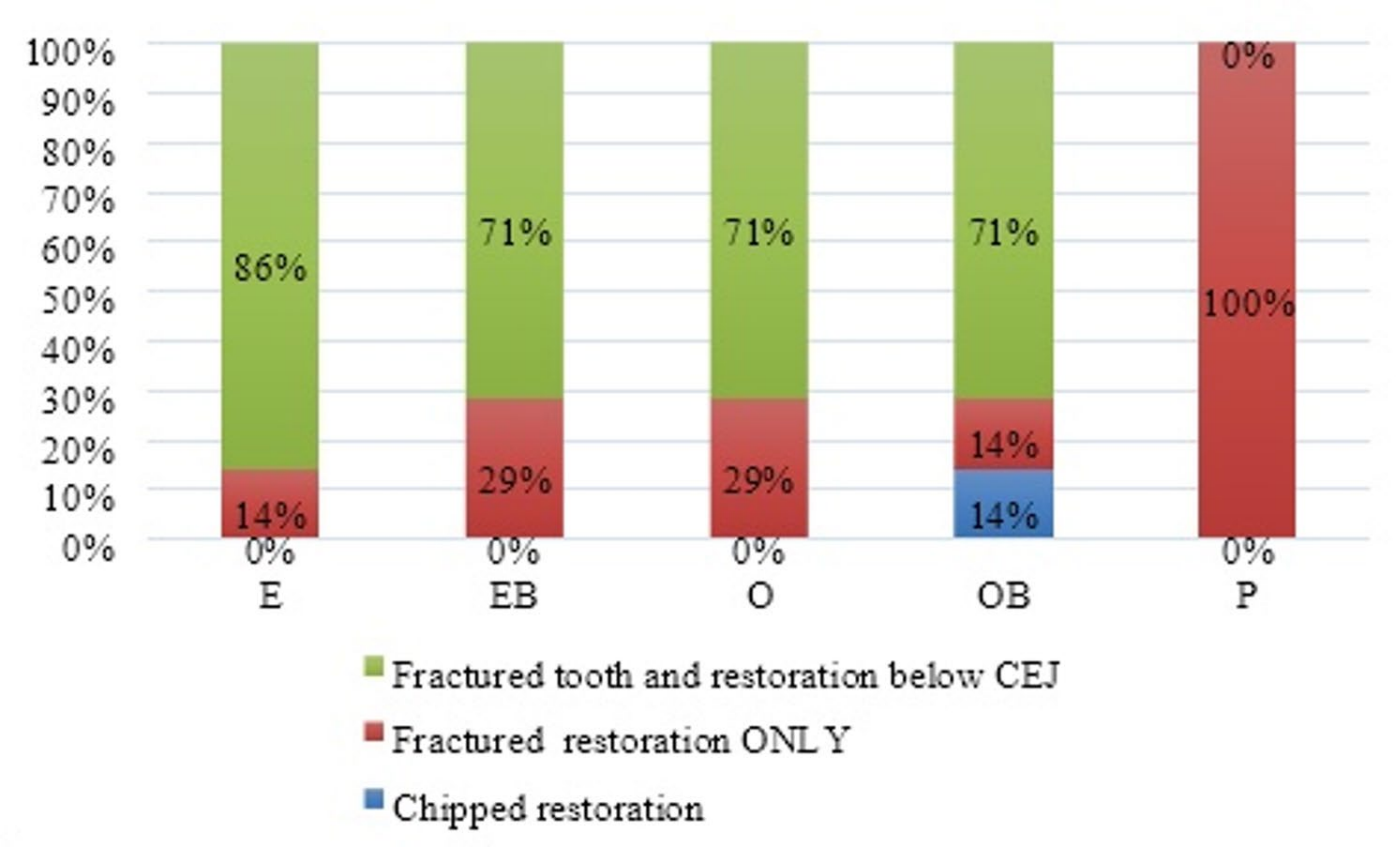
Stacked bar chart showing failure pattern distribution

All specimens of the P group expressed a favorable fracture. While for groups E, EB, O, and OB, the failure pattern was catastrophic in 86%, 71%, 71% and 71% of the specimens, respectively, Intergroup comparison and summary statistics for failure patterns are presented in Fig. 4.

**Fig. 5:**
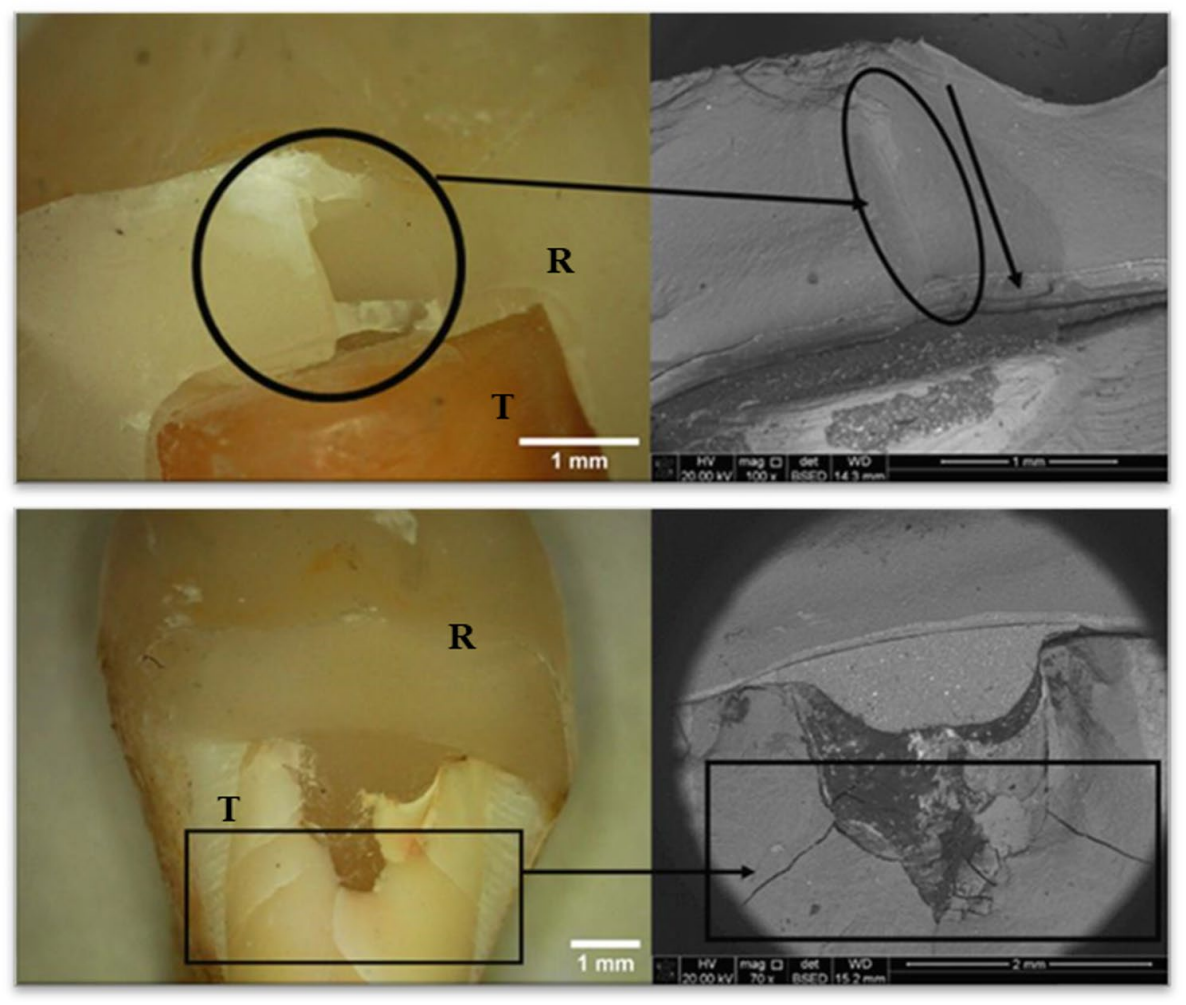
Representative stereomicroscope and scanning electron microscope (SEM) images illustrating favorable (repairable) fractures (upper row) and catastrophic (irreparable) fractures (lower row). R: restoration; T: tooth

**Fig. 6:**
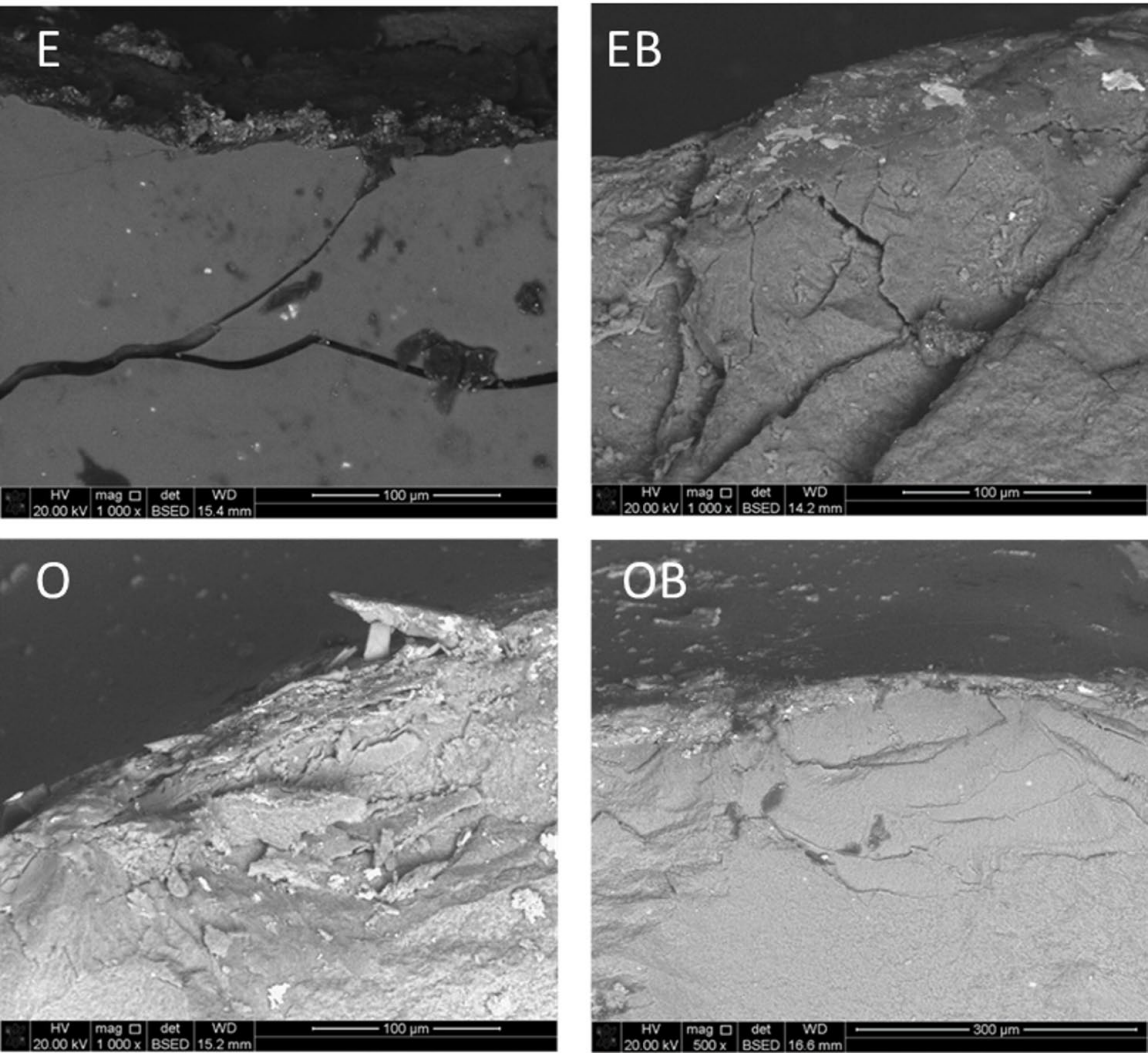
Higher-magnification SEM images illustrating catastrophic failure patterns in groups E, EB, O, and OB. Fractures are characterized by vertical wedge-opening cracks originating from the occlusal surface and progressing apically

Stereomicroscopic and SEM overview images (Fig. 5) were used to classify failure modes as favorable or catastrophic, while higher-magnification SEM images (Fig. 6) documented the apical extension of vertical wedge-opening fractures originating from the occlusal surface and progressing apically beyond the cemento-enamel junction, frequently resulting in palatal cusp splitting and severe root involvement.

**Fig. 7:**
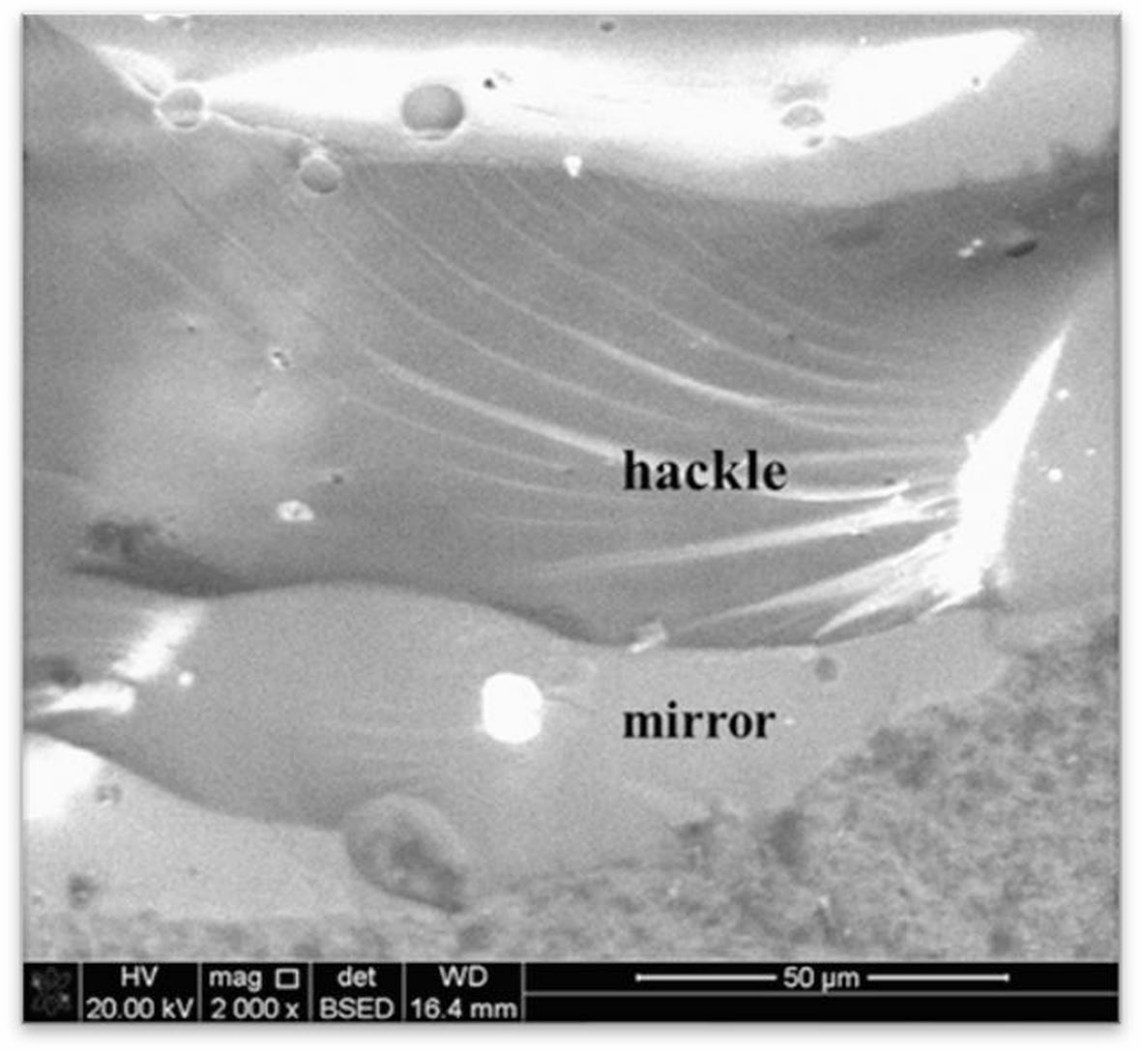
SEM micrograph illustrating fractographic features at the fracture origin, including a fracture mirror surrounded by hackle markings. Hackle directionality indicates crack propagation away from the origin, supporting localization of fracture initiation at the occlusal loading area

Fracture origins were identified by tracing crack paths backward from hackle toward mirror zones and compression curls. Fracture mirrors were identified as relatively smooth regions surrounding the crack origin and adjacent to hackle regions (Fig. 7). The mirror–hackle transition was used to confirm the location of crack initiation, as fracture mirrors represent the earliest stage of crack growth prior to acceleration [32, 33].

**Fig. 8:**
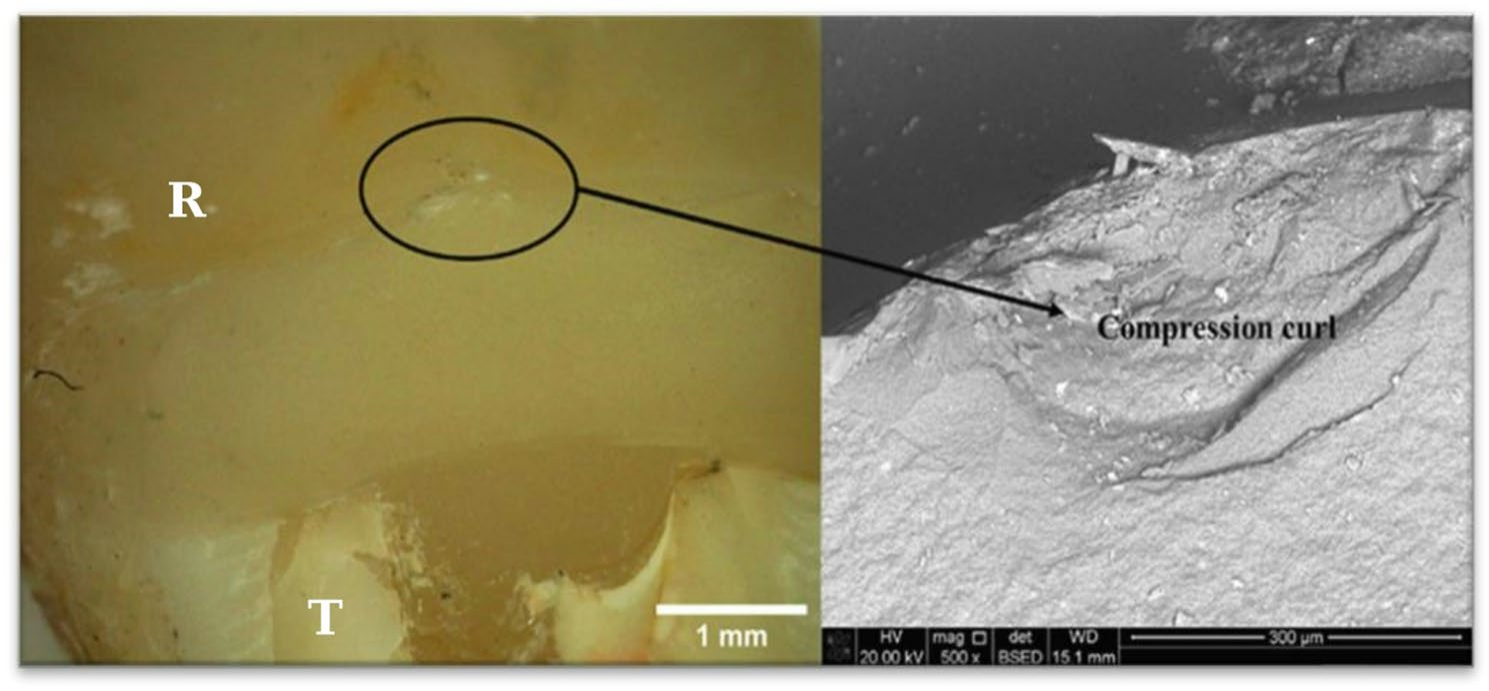
Major occlusal contact loading area, identified by the presence of a compression curl; R: Restoration, T: Tooth

Compression curls were consistently observed at the major occlusal contact loading area (Fig. 8) and were used to confirm the loading direction and stress state at failure. The presence and orientation of compression curls indicated compressive loading at the occlusal surface with tensile stresses developing on the opposing surface, supporting identification of the occlusal fracture origin [34, 35].

By integrating the directional information provided by hackle markings (Fig. 7), the localization of fracture mirrors (Fig. 7), and the confirmation of loading direction from compression curls (Fig. 8), the fracture origin was consistently traced to the occlusal loading area in all fractured specimens.

## Discussion

A high incidence of fractures of ETT, especially maxillary premolars, was reported in clinical practice and literature [15, 36]. According to previous studies, the typical biting force in the premolar region ranges from 222 to 445 N, with an average of 322.5 N. During clenching, occlusal forces may reach values as high as 520 to 780 N, with an average of approximately 660 N [37, 38]. Although the null hypothesis of this study was rejected because a statistically significant difference was found regarding the effect of different restoration approaches on both fracture strength and failure patterns of endodontically treated maxillary premolars, the mean fracture loads for all tested groups were higher than the maximum biting forces. As a result, all the treatment approaches should be able to withstand the masticatory forces.

The results of our study showed that the highest mean fracture strength was observed in Group P (1676.29 ± 191.25 N), followed by Group OB (1475.00 ± 198.72 N) and Group E (1374.71 ± 371.91 N), with no statistically significant difference in fracture strength among the three groups. This was in agreement with Elmashad et al. [39] who reported no statistically significant difference in fracture strength of endodontically treated maxillary premolars with endocrowns (1216.21 N), or post and core (1183.31 N). As a result, they recommended the realization of the less invasive option whenever possible.

The results observed in Group P may be partly attributed to the standardized all ceramic crown preparation with a controlled ferrule, which preserved sound cervical dentin and provided a bracing effect around the remaining tooth structure. Moreover, the use of glass fiber posts significantly enhanced the fracture strength of ETT in Group P. This effect may be explained by their low elastic modulus and the similarity in elastic moduli between glass fiber posts and natural dentin which permits flexural behavior under occlusal loading and promotes more favorable stress distribution to the cervical tooth structure, thereby enhancing resistance to masticatory forces [14].

Additionally, the use of bulk-fill composites in combination with fiber posts may have contributed to the improved fracture strength and more favorable failure patterns observed in endodontically treated premolars in the present study. This aligns with the findings of Spinelli et al. [40] who supported the use of this restorative combination, as it was associated with more favorable failure patterns. They recommended bulk-fill composite as a restorative material due to its ability to achieve higher average fracture strength, in addition to offering superior ease of use, making it a viable or even preferred option for restoring endodontically treated teeth.

In contrast, opposite results for post, core and crown approach were reported in the systematic review by Onofre et al. [41]. The poor performance of fiber posts was attributed to the absence of a ferrule and the preservation of only a single coronal wall. This highlights the influence of multiple factors on the survival and success of post-retained restorations in ETT, including the presence of a ferrule, the number of remaining coronal walls, and the type of post used [41].

Group OB showed high fracture strength (1475.00 ± 198.72 N), non-significantly different from that of conventional crowns, which may be related to the design of overlay restorations with buccal veneers, where complete coverage of the buccal surface redirected functional forces more favorably along the tooth’s long axis. Additionally, the presence of a foundation material with a low elastic modulus in the pulp chamber may have further contributed to force dissipation, enhancing structural integrity.

Supporting this, the findings of a study by Alassar et al. [42] who suggested that extending margins seems to have the effect of a ferrule, which resulted in their study in a better stress distribution. In addition, overwrapping or capping the cusp enhanced fracture strength [42], Though not directly addressing the same design but the importance of extended margins in overlay designs rather than the flat design was highlighted.

Endocrown design with butt joint showed positive results (1374.71 ± 371.91 N) compared to Group P and OB. A justification to this is that the butt joint design of endocrown provided an even, wide, stable surface of the tooth structure that resists the compressive stresses, because it is prepared parallel to the occlusal plane to ensure stress resistance along the main axis of the tooth, also even and increased thickness of ceramic used in the restoration enhanced the fracture load values [43]. But this adversely affected the failure pattern due to the geometric design of the preparation, which likely generated wedging forces on the tooth structure under occlusal loading, ultimately leading to catastrophic fracture.

For group EB (1047.41 ± 163.90), the results had no statistically significant difference with group E and group O (987.99 ± 125.01 N). This was not in agreement with Ahmed, et al. who compared the fracture strength of 3 different restoration approaches for root canal treated premolars; endocrown with flat occlusal table, endocrown with circumferential ferrule and endocrown with only buccal ferrule and in their study the endocrown designs with the buccal ferrule preparation revealed a significantly lower fracture strength (661 ± 143 N) in comparison to endocrown with flat occlusal Table (870 ± 167 N) and endocrown with circumferential ferrule (1225 ± 172 N) They explained their results by suggesting that in endocrowns with ferrule only on the buccal surface, the buccal wall of dentin is trapped between the pulpal inlay and the buccal margin of the endocrown, which might compromise the integrity of the buccal surface, making it more susceptible to weakening and leading to lower mean fracture strength values, especially with the absence of the reciprocal support of the lingual axial wall in addition to the wedging action of the intrapulpal extension of the restoration leading to more weakening. Nevertheless, the lowest mean fracture of the buccal ferrule group was still higher than the highest estimated occlusal chewing forces [29].

Regarding group O (987.99 ± 125.01 N), it showed comparable fracture resistance with group E and EB. The results of this study were partially in agreement with the findings of Abbas et al. [43] who compared the fracture resistance of overlays (1350 ± 82.202) with conventional post, core, and crown designs (2013.750 ± 100.915) and endocrowns (1079.375 ± 66.248) in maxillary endodontically treated premolars and reported a statistically significant difference between groups. They supported the use of overlay design when anatomic cusp reduction is used, confirming that adhesive cusp coverage restorations increase fracture strength. Also, favoring anatomic cusp reduction in overlay designs, Rocca et al. [35] suggested in their study that flat overlays retained solely by adhesive techniques are not suitable for restoring severely compromised premolars. Furthermore, adhesive cementation of flat restorations without any form of mechanical retention remains clinically challenging and is generally not recommended. In a study by Mosallam et al. [9], a statistically significant difference was recorded between overlays and endocrowns. The use of resin composite base material (925.55 ± 61.12 N) has improved the fracture strength of endodontically treated premolars than endocrown restorations (659.57 ± 79.91 N). Moreover, the addition of a polymerization modulator as the stress-decreasing resin (SDR) in bulk-fill flowable materials was proven to decrease the polymerization stress values by 60–70% in methacrylate and nano-hybrid flowable composites. The bulk-fill flowable material SDR was proven to lessen polymerization stress, cuspal deflection, and flexural modulus [44]. Additionally, as a material with a low modulus of elasticity it allows higher deformation under stresses thus dissipating the stress and improving the fracture strength [9]. The conflicting values with our study, especially for endocrown restorations, may have been attributed to the difference in ceramic material used. In their study, Vita Enamic hybrid ceramic was used for the endocrown design compared to higher fracture strength lithium disilicate in our study.

In this study, the majority of fractures occurred in the palatal region of the teeth. This is consistent with the findings of Ferri et al. [36] and Panahandeh et al. [45], who reported that the palatal cusp experiences greater stress compared to the buccal cusp. In light of this finding, it is advisable to consider additional reinforcement of the palatal cusp during restorative procedures. This may be achieved by optimizing the thickness of the restorative material in this area or by employing cuspal coverage designs. Particular attention should be given to the design and material selection for restorations to enhance the structural support of the palatal cusp and thereby reduce the risk of fracture under functional loading.

Mode of failure for group P (favorable) was statistically significant from other tested groups (Catastrophic). This may be due to the fiber post restorations which significantly increase the incidence of restorable fractures and were proven to be associated with absence of root fractures [14]. Favorable failures observed in Group P showed crack propagation limited to the crown without involving the underlying tooth structure in 100% of the samples. This finding aligns with a study by Gök et al. [46] which demonstrated that stabilizing the core and distributing functional loads along the root canal by the fiber post prevented the wedging effect commonly associated with post-less designs, ensuring that any potential failure remains coronal and repairable, thus preserving the tooth for future treatment. These results reinforce the clinical benefit of fiber posts in enhancing structural integrity of cervical dentin and achieving predominantly restorable fractures, which is essential for ensuring the longevity and predictability. In addition, the combined influence of post placement and a full-crown ferrule may have contributed to the improved fracture strength and favorable failure patterns observed. By encircling and reinforcing the cervical dentin, the ferrule effect can reduce stress concentration at the core–tooth interface and help limit crack initiation and propagation [29].

As a result, it is not possible to fully separate the reinforcing effect of the post from the structural advantage conferred by the ferrule. This potential confounding should be considered when interpreting the results, and caution is warranted in attributing the mechanical performance solely to the post itself [46].

Furthermore, Liu et al. [47] highlighted the significant influence of the elastic modulus of the core foundation on the fracture behavior of all-ceramic crowns. They concluded that the failure mode of porcelain crowns is affected by the stiffness of the supporting core material. Accordingly, the porcelain crown fractures observed in this study may be partly attributed to the low elastic modulus of the composite resin core (approximately 16 GPa).

Catastrophic failure in partial coverage designs may also be related to restoration material where lithium disilicate ceramics are rigid materials with high stiffness lacking the tendency to bend under stress with resultant stress concentration in the residual tooth structure and catastrophic types of failures [48]. This finding was in accordance with previous studies [38, 49], which reported that the failure patterns observed with composite endocrowns were more favorable compared to those of lithium disilicate ceramics, as the latter often involved root fractures leading to unrestorable teeth. Also, Zhu et al. [50] recorded in their study poor survival rates in terms of tooth preservation in association with ceramics compared to resin composite in restoration of maxillary premolars. They reported that increasing the elastic modulus of the material may be beneficial to bond durability between the endocrown and the tooth but on the other hand, it transfers stresses to residual tooth structure with increased irreparable failures [51].

Qualitative SEM fractographic examination has been widely used in dental materials research to identify fracture origins and crack propagation features, and has been shown to reliably distinguish between different modes of failure and to correlate well with loading conditions in vitro. Recent studies continue to apply SEM analysis to characterize failure mechanisms of ceramic and composite restorations, demonstrating that features such as compression curls, hackle, and mirrors provide mechanistic insight that complements mechanical testing data [34, 52].

Fractographic analysis in the current study revealed that the fracture origin in all specimens was located at the point of load application occlusally. This finding aligns with several studies reporting an occlusal-to-apical direction of crack propagation underneath the loading ball revealing stress concentration that triggered the failure [35, 53, 54]. Different fracture origins were reported in literature pointing to design inconsistencies (e.g., feathered or uneven margins) that increase fracture risk [55, 56]. In Øilo and D Quinn study [57], the most common fracture origins were marginal defects including thin, chipped, cracked or uneven crown margins. In our study Feather-edge or sharp margins were avoided while smooth and moderately thick crown margins were adopted to improve the restorations durability. The predominance of vertically oriented cracks progressing from the occlusal surface toward the root indicates a tensile-driven brittle fracture mechanism governed by stress concentration beneath the loading contact, rather than margin-initiated or adhesive interface failure. indicating that restoration design and loading configuration, rather than interfacial defects, primarily influenced the fracture behavior under the conditions of this study [34]. This interpretation is consistent with contact-damage models for ceramic and tooth-restoration systems subjected to high-magnitude occlusal loading [49].

Small fracture mirrors were identified as relatively smooth regions surrounding the fracture origin. These mirrors represent the area where a crack rapidly radiates outward from the point of initiation. Locating the fracture mirror is essential, as it provides insight into the fracture origin, and its size reflects the stress level at the moment of failure. Specifically, a smaller mirror indicates higher stress at the origin site, suggesting a structurally strong component and/or the presence of a minor strength-limiting flaw. Conversely, a larger mirror implies a lower failure stress, often associated with a weaker microstructure or a larger defect [32]. Hackles were also observed in the fractographic images. These features typically develop in response to tensile stresses, confirming that the failure was brittle in nature and driven by tension. This finding highlights the importance of enhancing material toughness, crack resistance, and surface integrity and provides mechanistic insight into the observed failure pattern.

Crack propagation was reconstructed by identifying the directionality of hackle markings, which radiate away from the fracture origin, and by locating fracture mirror regions that represent the initial smooth zone surrounding the point of crack initiation. The presence and orientation of compression curls at the occlusal contact area were used as primary indicators of the loading direction and tensile-compressive stress distribution. By sequentially following these features from peripheral roughened regions toward smoother mirror zones and compression curls, the fracture origin was consistently localized to the occlusal loading site [34, 35].

Clinical decision making in the restoration of endodontically treated maxillary premolars should integrate both fracture strength and the nature of failure patterns. In the present study, post, core, and crown restorations exhibited the highest fracture strength and the most favorable, predominantly repairable failure patterns among the evaluated restorative strategies. Overlay restorations combined with a buccal veneer demonstrated fracture strength comparable to post-retained crowns while adopting a more conservative approach; however, this design was associated with a higher incidence of catastrophic, non-repairable failures. Although conservative adhesive restorations preserve greater amounts of tooth structure, their mechanical performance and failure behavior should be carefully weighed against functional demands. Accordingly, restoration selection should be based on a balanced assessment of remaining tooth structure, expected loading conditions, and anticipated failure characteristics, rather than fracture resistance alone.

Double-rooted maxillary first premolars were selected because their distinctive anatomy further compromises fracture resistance. A pronounced mesial root concavity and a palatal radicular groove on the buccal root create areas of stress concentration and structural weakness, predisposing these teeth to cusp fracture, wedging under occlusal loading, and root splitting. Moreover, their reduced cervical dentin thickness, an area critical for stress transmission which facilitates the initiation and propagation of vertical root fractures [2].

The overlay group (O) was designed with full occlusal coverage of both buccal and palatal cusps. This approach follows established protocols for reinforcing endodontically treated teeth with extensive structural loss and aligns with recent findings that complete coverage provides superior protection against catastrophic fracture compared to functional-only reduction [58].

SDR bulk-fill composite was selected due to its stress-decreasing properties and relatively low elastic modulus, which reduce polymerization shrinkage stress and cuspal deformation. This allows better stress dissipation under occlusal loading and provides a favorable biomechanical foundation for adhesive partial-coverage restorations [59].

It is important to acknowledge that the present study does not fully replicate dynamic intraoral conditions. Although this represents a limitation, the use of static loading allows a controlled assessment of the ultimate strength and failure threshold of both the restorative materials and the tooth structure. Fracture under static loading typically initiates at predictable sites of stress concentration near the point of load application, enabling consistent comparison among different restorative designs and provides a controlled measure of structural performance without the confounding effects of time-dependent factors such as viscoelastic behavior, microcrack propagation, cyclic fatigue, thermocycling, or oblique loading.

Our results represent the maximum structural threshold of the tested designs which exceeded reported physiologic masticatory forces (≈ 445 N) and may reach up to ~ 780 N during clenching. While the findings provide valuable insights into relative fracture strength and failure patterns, the clinical performance of these restorations may differ due to the influence of repeated functional loading, thermal stresses, and patient-specific occlusal conditions.

In this study, vertical loading was selected to assess the ultimate load-bearing capacity and to allow standardized approach to evaluate relative differences in fracture strength and failure patterns among restorative designs. While oblique loading may better simulate parafunctional or group-function occlusion, it introduces additional variables related to cusp inclination and contact location. Since lateral forces were not applied, the results should be interpreted as a measure of structural resistance rather than a comprehensive assessment of clinical retention. Further studies involving oblique loading are recommended to fully simulate the oral environment and enhance clinical applicability.

Future direction in this field could explore other materials that combine the best properties of different materials, whether it concerns posts or coronal restorations. Research on posts should focus on techniques that would not necessitate additional canal preparation. More randomized clinical trials should be conducted to assess the long-term survival of endodontically treated premolars restored using minimal intervention endodontic-restorative concepts and techniques.

## Conclusions

This study highlights the importance of selecting appropriate preparation designs to optimize the mechanical performance and clinical success of restored endodontically treated premolars.

In the essence of this study, these conclusions could be established:

1. The post, core, and crown restorations, and overlays with buccal veneer demonstrated comparable fracture strength for endodontically treated upper premolars.
2. Endocrowns with buccal veneer and overlays showed the lowest fracture strength.
3. The post, core, and crown showed the most favorable failure pattern.
4. The tested designs withstood loads exceeding physiologic masticatory forces, indicating high structural capacity. However, clinical interpretation should be cautious, as cyclic fatigue, parafunctional activity, and non-axial loading were not simulated.

## Data Availability

All relevant datasets and their supporting information files generated and analyzed during this study are available from the corresponding author upon reasonable request.

## Abbreviations

ETT: Endodontically treated teeth
SDR: Stress-decreasing resin
CAD: Computer-aided designing
CAM: Computer-aided milling
SEM: Scanning electron microscope
